# Treatment of aortic aneurysms in elderly patients

**DOI:** 10.1186/1471-2318-11-S1-A66

**Published:** 2011-08-24

**Authors:** G Vigliotti, A D’Alessandro, G Fornino, A E Puca, G Capparelli, M Cuofano, A Naddeo, L Brunico, R C Vigliotti, G Accarino

**Affiliations:** 1 Chirurgia Vascolare ed Endovascolare, Azienda Ospedaliera Universitaria “OO.RR. San Giovanni di Dio e Ruggi d’Aragona”, Scuola Medica Salernitana, Salerno, Italy

## Background

The treatment of aortic aneurysms in elderly patients, especially those aged around 80, has undergone a significant change with the introduction of aortic stent (EVAR). Although some studies are in progress regarding the improvement of long-term results with regard to "open" surgery, there is no doubt that the exclusion of the aneurysm with a stent is more acceptable in terms of the immediate patient management, morbidity and mortality. In our institution we have had a significant increase in the treatment of patients with aortic aneurysm treated with EVAR. We present data for the last two years 2009 and 2010.

## Materials and methods

In our Centre, from January 2009 to November 2010, 168 aneurysms of the thoracic and abdominal aorta were treated, of which 135 were elective and 33 emergency. Of these 39 % were patients over 75 years of age. In 2009 we operated 52 patients of whom 7 (13:45%) for thoracic aortic aneurysm and 45 (86.55%) for aneurysms of the abdominal aorta. 6 thoracic aneurysms (86%) were operated on electively and 1 (14%) urgently, all with a stent implant. 40 aneurysms of the abdominal aorta (89%) were operated on electively (of these 25 (62.5%) with EVAR and 15 (37.5%) with "open" surgery) and 5 (11% ) in emergency (all in "open"). In 2010, 116 patients were treated, of which 20 (17.3%) had an aneurysm of the thoracic aorta and 96 (83.4) an aneurysm of the abdominal aorta. 14 aneurysms of the thoracic aorta (70%) were operated on electively and 6 in emergency (30%), all with EVAR. 80 aneurysms of the abdominal aorta (83.3%) were operated on electively (of these 70 (87.5%) with EVAR and 10 (12.5%) treated with "open" surgery) and 16 (16.6%) in urgency (two of these (12.5%) with EVAR and 14 (87.5%) treated with "open" surgery).

## Results

The mortality rate in elective patients was 0.7% (1 patient underwent an endograft for abdominal aorta), while in emergency mortality was 18% (6 patients all operated in "open"). In one patient who underwent aortic stent there was an acute renal failure with permanent dialysis. Cardiological problems were resolved before discharge.

## Conclusions

The treatment of aneurysms of the thoracic and abdominal aorta has improved in terms of mortality and morbidity after the introduction of EVAR. In our experience, which tends to expand the use of aortic endoprosthesis, there are no substantial differences in outcome between EVAR and "open" surgery. Elderly patients maintain better treatment with an aortic stent graft.

## References

[B1] Nano G. Bianchi P Trattamento endovascolare di Aneurismi dell’aorta addominale in pazienti ottantenari. Risultati a medio termine Ann Ital Chir 2008 79 335 340 19149361

[B2] Ballotta E Da Giau G Open abdominal aortic aneurysm repair in octogenarians before and after the adoption of endovascular grafting procedures-J Vasc Surg 2008 47 23 30 10.1016/j.jvs.2007.08.054 18060731

[B3] Mainente A Nano G Tealdi DG Il destino dell’ottuagenario operato di aneurisma dell’aorta addominale (Studio retrospettivo) It J Vasc Endovas Surg 1999 6 181 6

